# Tuning cell migration: contractility as an integrator of intracellular signals from multiple cues

**DOI:** 10.12688/f1000research.7884.1

**Published:** 2016-07-26

**Authors:** Francois Bordeleau, Cynthia A. Reinhart-King

**Affiliations:** 1 Meinig School of Biomedical Engineering, Cornell University, Ithaca, NY, USA

**Keywords:** Contractility, Crosstalk, cell migration, focal adhesions

## Abstract

There has been immense progress in our understanding of the factors driving cell migration in both two-dimensional and three-dimensional microenvironments over the years. However, it is becoming increasingly evident that even though most cells share many of the same signaling molecules, they rarely respond in the same way to migration cues. To add to the complexity, cells are generally exposed to multiple cues simultaneously, in the form of growth factors and/or physical cues from the matrix. Understanding the mechanisms that modulate the intracellular signals triggered by multiple cues remains a challenge. Here, we will focus on the molecular mechanism involved in modulating cell migration, with a specific focus on how cell contractility can mediate the crosstalk between signaling initiated at cell-matrix adhesions and growth factor receptors.

## Introduction

Cell migration is critical to numerous physiological and pathological conditions, including development, wound healing, and tumor cell metastasis. Cell migration within an organism is seldom solely random but rather is in response to a directed set of signaling cues. Even during diseases such as cancer, migrating cells appear to follow guiding signals ^1, 2^. The cell microenvironment can provide and support a multitude of different kinds of cues that trigger and direct cell migration ^2– 5^. In turn, cell migration requires complex, regulating intracellular machinery to accurately respond to the cues and properly interact with the extracellular matrix (ECM). This is highlighted by the fact that not all cells possess the same migrating potential nor do they respond to a given cue in the same way ^6– 9^.

The ability of adherent cells to migrate is dependent primarily on their ability to dynamically regulate integrin-mediated cell-ECM linkages at specialized focal adhesions (FAs) and hemidesmosome membrane domains ^10– 12^. The mechanisms and dynamics behind FA formation and maturation are increasingly well documented in both two-dimensional (2D) and three-dimensional (3D) matrices ^12– 18^. Given our present knowledge, these mechanisms appear to be shared by most adherent cells.

Directed cell migration requires proper inputs. These migration cues include biochemical cues present in the form of soluble growth factors and hormones, direct physical stimulation such as shear flow or electrical fields, and the cues arising from the physical and architectural organization of the ECM ^19^. However, a current limitation in our understanding of the true impact of these cues lies in the fact that they are largely studied independently from each other, and most findings are generally cell type specific. There have been some efforts to combine at least two different migration cues ( 20– 22, and reviewed here ^19^). Additionally, it is not clear why, in a given cell population, cells can exhibit different migration behaviors despite using the same machinery and being subjected to the same pro-migration cues.

Here, in an attempt to explain the migration differences that exist between cells exposed to the same cues, we will explore the major molecular features that finely regulate cell migration. We will first present an overview of the major factors and cues, both extracellular and intracellular, involved in controlling cell migration. We will then focus on mechanisms that can finely tune cell migration for a given set of migration cues and discuss how cell contractility may play a central role in the integration of intracellular signals.

## Determinants of cell migration

Given a specific set of extracellular cues, cell migration is typically a function of the nature (quantity, presentation, and so on) of those cues. Notably, growth factor stimulation can trigger cell migration, and the presence of concentration gradients can enable directed migration through chemotaxis ^19, 23^. Chemotaxis is a major driver of cell movement and is instrumental during development and for angiogenesis in tumors ^4, 23^, for example. Since not all cell types respond to the same set of given cues, genetic differences between different cell types may readily explain why some cells have increased affinity for specific chemical cues. For instance, differential expression of growth factor receptor families, including their various isoforms, can prime cells to respond to a specific subset of growth factors. An example is the vascular endothelial growth factor receptor (VEGFR) family, which is normally expressed in cells of vascular origin and where both VEGFR1 and VEGFR2 are potent inducers of endothelial cell migration ^24^. Importantly, however, differential expression of growth factor receptors does not always correlate with cell migration during experimental observation ^7, 8^. A striking example is when comparing the migration induced by different growth factors of highly invasive MDA-MB-231 and the weakly metastatic MCF7 cell lines. For instance, stimulation with the insulin-like growth factor 1 (IGF-1) triggers more potent migration in MDA-MB-231 cells compared with MCF7 cells, and the effects are reversed when epidermal growth factor is used instead ^7^. Estrogen receptor-positive tumor cells, such as MCF7 cells, are characterized by overexpression of the IGF-1 receptor ^25^, whereas MDA-MB-231 cells are known to overexpress EGFR ^26^. Moreover, although the average response of a cell population to growth factor stimulation provides critical information when comparing different cues or cell types, it does not explain the variability between individual cells within a cell population. In fact, not all cells within a population will move efficiently toward the source of a gradient; some cells will not move at all or will go in the opposite direction ^27, 28^. Therefore, differences in the expression patterns of growth factor receptors are not necessarily sufficient to explain the differences in cell migration observed experimentally.

In comparison with soluble growth factors, migration cues arising from the ECM have traditionally been more difficult to study given the inherent engineering challenge of properly recreating a physiologically relevant ECM scaffold ^29, 30^. Nevertheless, several groups, including our own, have over the years made several breakthroughs in understanding how the physical properties of the ECM can influence cell migration and provide migration cues ^3, 17, 31, 32^. Some of the early seminal work in this area has been instrumental in showing that cell migration is guided along matrix stiffness heterogeneities and gradients ^3, 33^. Importantly, recent advances in our ability to control the ECM architecture and its mechanical properties have enabled increased scrutiny of cell migration, especially in the 3D microenvironments ^34– 36^. Notably, work within the field has addressed how the 3D architecture of the ECM, including the presence of microtracks or steep ECM interfaces, can impede, facilitate, or provide contact guidance for cell migration ^17, 37– 40^. The various kinds of physical features present in the ECM and their impact on cell migration have recently been reviewed ^5, 41^. Interestingly, the presence of local heterogeneities in the microarchitecture of the ECM could help explain in part the migration difference between individual cells within a cell population.

Working in concert with the physical ECM and microenvironment, adhesion molecules are the central regulators of cell migration since they are the linkage between the cell and the supporting ECM ^12^. Interestingly, similar observations have been made regarding the expression of cell adhesion proteins, such as the integrin receptors, as with growth factor receptors. That is, integrin expression is not always correlated with migration response and exhibits cell-type-dependent variations ^42^. Integrins are αβ heterodimers and their ligand binding specificity is determined by which integrin subtypes assemble to form a pair (for example, α5β1, αvβ3, and α4β1 for fibronectin; α1β1 and α2β1 for collagen; and α2β1, α3β1, and α6β4 for laminins) ^12^. Given their large diversity (18α and 8β in 24 different possible pairs) ^43^, it is easy to assume that they may play a vital role in differentially regulating cells’ migration ability. Indeed, in some cases, differential expression of integrin subtypes, such as β3, correlates with altered cell migration potential ^44, 45^. However, this correlation does not hold universally true. For instance, different tumor cell lines can have similar levels of integrin expression and different migration potential ^46– 48^. There are also examples where cell migration can be regulated by controlling the activation state of integrins or the integrin-mediated downstream signaling ^13, 49– 51^. The signaling initiated by integrins controls several key processes required for migration, including FA turnover and control of actin dynamics ^52– 55^. Notably, the integrin β1 plays a central role in regulating FA dynamics through the activation of the Src/FA kinase (FAK) pair at FAs, which in turn regulates Rac1 and RhoA at the cell front ^55– 57^. Interestingly, the mechanical properties of the cellular environment regulate an integrin β1-mediated activation of specific FA proteins, including FAK ^13, 15, 58– 60^. Increasing the stiffness of either 2D or 3D matrices results in higher FAK phosphorylation levels as well as larger FAs in several cell types ^58, 60^. In addition, these signaling pathways are regulated by both feedback loops and crosstalk with other transmembrane receptors ^61– 64^.

Therefore, when these results are considered together, migration differences between cells cannot be explained solely by differential expression of the receptors associated with a given cue. In fact, one explanation for why cells display a wide range of migration behaviors may reside in their ability to integrate the different intracellular signals triggered by the available migration cues they experience. In this context, it becomes crucial to better examine the mechanisms that can modulate the signaling triggered by migration cues.

## Integration of intracellular signals through cell contractility

**Figure 1.**
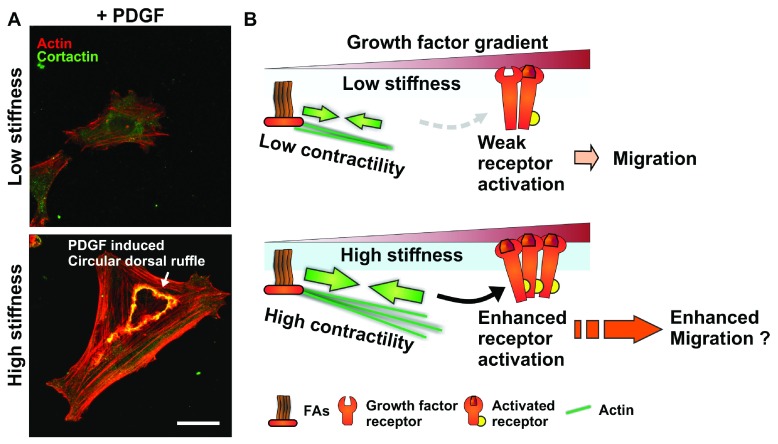
Matrix stiffness-mediated regulation of growth factor activation as a modulator of cell migration. ( **A**) Matrix stiffness regulates the formation of actin and cortactin-rich circular dorsal ruffle structures downstream of platelet-derived growth factor (PDGF) receptor activation in response to PDGF stimulation in smooth muscle cells (low stiffness: 1 kPa; high stiffness: 30 kPa; see 70 for details). Scale bar is 20 μm. ( **B**) Schematics of proposed signaling crosstalk between focal adhesion (FA) and growth factor receptors depicting how increased matrix stiffness could potentiate growth factor-induced signaling, resulting in increased cell migration.

The ability of cells to integrate cues from multiple cues has been studied for many years, often with an emphasis on transmembrane receptor interactions and crosstalk in specific cell membrane domains ^61, 62, 65, 66^. Among the best known examples of this are the interactions and crosstalk between integrins and various growth factor receptors ^61– 64^. EGFR and VEGFR, for example, point to an integrin-mediated role in efficient activation of these receptors ^61, 63, 67– 69^. However, an emerging concept is that integrin-growth factor receptor crosstalk is facilitated by cell contractility ^68, 70, 71^. The consequences of this contractility-mediated crosstalk have important ramifications for the ability of cells to integrate migration cues like soluble growth factors and ECM stiffness. It is established that cell contractility is modulated by ECM stiffness ^68, 72^. For example, increasing ECM stiffness leads to both enhanced smooth muscle cell response to platelet-derived growth factor ^70, 71^ (see example in Figure 1A) and epithelial cell response to EGF ^68^. Interestingly, the interplay between ECM mechanical cues and growth factor signaling appears to be mediated in part by cell contractility. When cell contractility is inhibited through inhibition of the Rho-ROCK-MLC axis, cell response to growth factors is suppressed. Moreover, cells within a population tend to display a wide range of measureable contractility for a given ECM stiffness ^73, 74^, which in turn could influence how sensitive each individual cell is to growth factor stimulation. A similar explanation could apply to various cell types that show different contractility levels ^68, 74, 75^.

In addition to being found in the soluble microenvironment, growth factors can be found bound to the matrix and are in fact not always readily accessible ^65, 76, 77^. Transforming growth factor-beta (TGF-β) is an interesting example. It is normally found encapsulated within a latent TGF-β1 complex bound to the ECM ^78^. This complex contains an RGD sequence that enables integrin binding, and cells capable of generating sufficient force will ultimately be more able to access active TGF-β1 ^79^. Moreover, the underlying ECM needs to be sufficiently stiff to support the force required to unwind the latent TGF-β1 complex, which would otherwise deform, dissipating the force without releasing the stored TGF-β ^79^. Overall, these results suggest that cell migratory response to growth factor cues may in fact be ultimately linked to the interplay between ECM stiffness and the contractile state of the cell ( Figure 1B).

An interesting prediction arising from the studies described above is that cells on stiffer substrates could exhibit increased migratory response in the presence of a growth factor gradient in a process that could be cell type specific. There has been some work using numerical modeling of the integration of migration cues to predict migration response; however, these models contain the assumption that the cues are purely additive from the perspective of the cell ^80^. However, they do not address how each of these cues could synergize or interfere with one another since their work is based on experimental data where each of these cues is studied in isolation ^81, 82^.

Indeed, one of the challenges in this work is to assess whether cues are additive or whether the signaling triggered by one cue alters the response to the other cues. Notably, different migration cues can either directly compete or cooperate with each other ^20– 22, 83^. Work done to elucidate the link between the ECM and growth factor stimulation can provide some mechanistic insight into how different cues may lead to completely different biological outcomes. For example, there is a critical ECM stiffness at which TGF-β1 stimulation switches from being pro-apoptotic to pro-epithelial to mesenchymal transition ^84^. ECM stiffness can also promote an EGF-dependent change toward a “malignant” phenotype in mammary epithelial cells ^58, 68^. Interestingly, the phenotypical changes observed in mammary cells in response to ECM stiffness and EGF were dependent on FAK and ERK co-activation, whereas altering cell contractility state overrides the system ^58, 68^. In this context, there are likely regulating signaling components, particularly those modulating both the cell contractile state and cell interactions with the ECM, that ultimately play a central role to integrate various extracellular cues.

## Tuning cell migration by modulating focal adhesion signaling nodes

With regard to the signaling that controls cell migration and contractility, most of the work that has been performed has focused on key components that are ubiquitously expressed in almost all cells. These key components are usually master regulators that act as an on/off switch such as the Rho GTPases as well as FAK, PI3K, and Src family kinases ^57, 85– 90^. Importantly, though, both proper activation and localization of these proteins are instrumental in modulating cell migration ^13, 85, 91– 93^. One way to regulate the FAK/Src signaling initiated at FAs is through feedback loops ^64^. For example, members of the protein kinase C (PKC) family can be activated upon cell adhesion to fibronectin along with FAK; in turn, increased PKC activity leads to increased activation of α5β1 integrins and cross-activation of the α4β1 integrins, essentially leading to more FAK activation in a feed-forward loop ^64^. It is interesting to note that such PKC-mediated integrin regulation exists in several other cell types. Notably, PKCε positively regulates integrin-dependent cell migration in gliomas cells, whereas PKCα plays an opposite role ^94^. Study of renal carcinoma cell migration suggests instead a PKCδ-dependent mediation ^95^. In addition, PKCs are often found downstream of growth factor receptor activation ^96– 99^. Notably, these studies also suggest that PKC isoform-mediated cell migration regulation is cell type dependent, even though PKC isoforms are roughly expressed at comparable levels in most tissues (GeneAtlas U133A, gcrma ^100, 101^). Therefore, there must exist cellular mechanisms to enable fine-tuning of cell migration by the various PKC isoforms.

**Figure 2.**
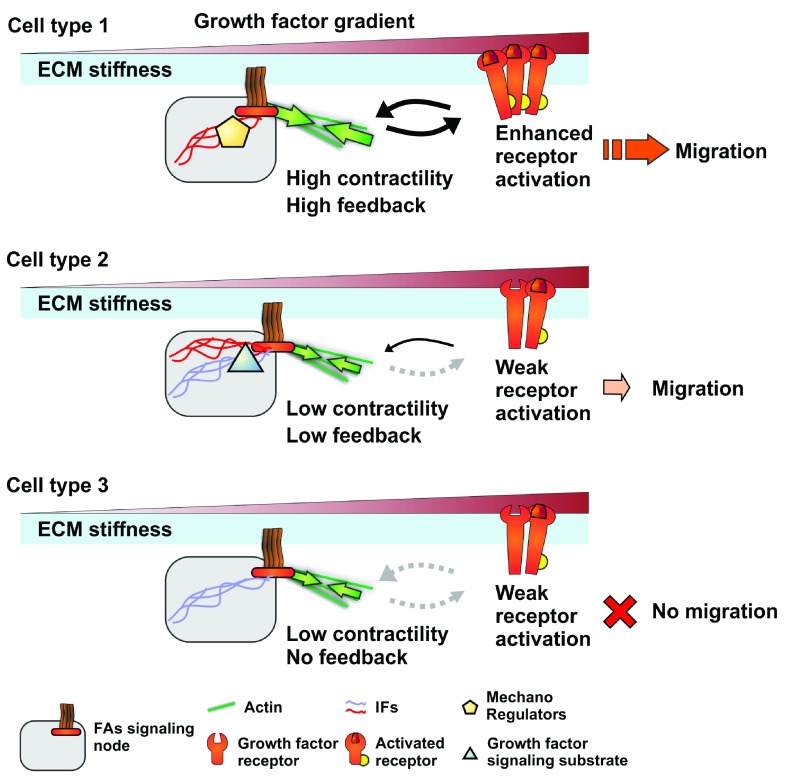
Focal adhesion (FA) signaling nodes and their potential role in modulating response to growth factors and subsequent cell migration. Schematics showing how the expression of different forms of mechanoregulating and scaffolding proteins in three cell types can influence intracellular signal integration by altering signaling nodes. Differential expression of these factors can alter the contractile state of the cell or directly enhance the feedback between growth factor receptors and FAs, resulting in a modulated response to growth factor stimulation that could regulate migration. These mechanoregulators can be structural and scaffolding proteins, such as different intermediate filaments (IFs), or signaling kinases, such as protein kinase C, or both. ECM, extracellular matrix.

Indeed, the ability of PKCs to modulate cell adhesion and migration depends on their association with intermediary proteins. For example, the receptor for activated C-kinase (RACK1) enables the interaction of PKCε with β1 integrin to promote glioma cell adhesion and migration ^94^, and PKCs can also modulate Src activity via RACK1 ^102^. In addition, PKC-mediated modulation of cell migration can be further tuned by the intermediate filament (IF) cytoskeleton expression profile of cells ^13, 103^. Notably, PKCε mediates integrin recycling and is required for efficient migration when cells express vimentin ^103^, an IF found in cells of mesenchymal origin and highly aggressive carcinomas ^104^. Alternatively, the expression of the keratin 8/18 IF pair, which is a hallmark of all simple epithelia, enables more efficient PKCδ activation of FAK, which in turn promotes more directed cell migration ^13^. Of note, the absence of the keratin 8/18 IF appears to alter the formation of the PKC/RACK1 complex, where PKCδ is replaced by PKCα ^13^. Most interestingly, recent work has shown that Rac1-mediated phosphorylation of the myosin heavy chain IIa at the front of the cell and in FAs is PKC dependent ^105^. It also appears that this specific myosin heavy chain phosphorylation mechanism helps to regulate both cell migration and mechanosensing ^105^. Moreover, we and others have recently shown that IFs are important modulators of cell contractility ^73, 106– 108^. Therefore, small differences in FA signaling node organization might be sufficient to alter the crosstalk between integrins and growth factor receptors and explain the diversity of cell-type-specific response to migration cues ( Figure 2). Overall, understanding how these scaffolding components can modulate major signaling pathways may provide key insights into cell-type-specific response differences to migration cues.

## A role for epigenetics and microRNA regulation in fine-tuning cell migration

An emerging and exciting area in cell migration research is how epigenetics may tie into the regulation of cell migration and the integration of different migration cues. Protein expression control through alternative splicing appears to be one such mechanism. Alternative splicing is a primary source of protein diversity, where one gene can generate multiple different versions of a protein ^109, 110^. Several proteins that possess alternatively spliced variants are linked to cell migration, including signaling proteins (such as β1 integrin, Rho GTPase, FAK, and PKCs) ^111– 113^, scaffolding and structural proteins (plectin, cortactin, and p130CAS) ^114– 116^, growth factors (TGF-β and VEGF) ^117, 118^, and ECM components (fibronectin) ^119^. The alternate versions of these proteins can have distinct functions compared with their canonical counterpart, resulting in altered cell behaviors, including migration ^115, 117^. Therefore, alternative splicing could play an important role in modulating migration cues and signal integration by altering signaling nodes and migration effectors. Interestingly, our recent work indicates that the interplay between matrix stiffness and cell contractility regulates alternative splicing of proteins produced by the cell ^120^. The implications of these findings with regard to signal integration of multiple cues are potentially significant since they suggest that cells have a self-tuning mechanism. Another highly relevant epigenetic mechanism worth exploring is the involvement of microRNA (miRNA). Indeed, recent work has linked the expression of several miRNAs to the ability of cells to integrate ECM mechanical cues as well as regulate cell migration ^121, 122^. Both of these aspects remain largely understudied and underappreciated.

## Designing experimental approaches to resolve signal integration to multiple cues

Our ability to move forward in the study of systems that combine multiple cues relies on our ability to engineer devices that can dynamically combine these cues in a controlled manner so that the cell response to these cues can be analyzed. A number of groups have designed such devices, where they recapitulate some of the characteristics of the ECM or the cellular microenvironment, the presence of chemical cues, or even cell-cell interactions ^27, 83, 123^. These engineered approaches range from simple tuning of ECM mechanical features ^70, 84^ to microfluidic devices that allow for the integration of multiple cues ^27, 83^. However, the numerous crosstalk and compensatory mechanisms that are likely triggered by multiple simultaneous input cues make it difficult to dissect the relevant signal transduction pathways, especially for signaling proteins that are involved in multiple pathways. Even stimulation from a single input can activate numerous different signaling pathways that are dependent on protein expression levels or the presence of point mutations ^13, 96, 124^. Therefore, a more integrative approach to study cellular systems in the presence of multiples migration cues should include temporal and spatial analysis of the multiple signaling nodes in cells as well as cell contractility and downstream migratory events. Importantly, determining how and when different compensation mechanisms get activated when cells are subjected to multiple cues is critical to understanding intervention points for drug therapies.

Interestingly, it is possible to dissect dynamic cellular responses to different perturbations by using a time series modeling approach ^125^. This particular modeling approach yields information on how and when cells can switch between different phenotypic states. Such an approach permits multiple inputs and output states, allowing a more holistic characterization of how cells integrate signals from multiple cues. A clustering approach can also be used to analyze signaling networks in cells subjected to different stimuli to help distinguish between shared pathways between receptors and reveal the response of specific classes of receptors to soluble factors ^126^. In addition, modeling approaches of signaling networks in the form of regulatory circuits can account for coupling and compensatory mechanisms ^127^. For example, modeling of regulatory circuits can describe the contribution of miRNAs and transcription factors in the coupling of signaling networks and can predict the migration mode adopted by tumor cells ^128^. Therefore, these modeling approaches are powerful tools to help identify compensatory mechanisms occurring in experimental systems and adequately describe the different possible states within a cell population.

## Summary

Although observations described in the literature can help explain how cell migration can be more finely tuned depending on the cellular and microenvironmental context, they do not yet offer a complete picture of how cells integrate various migration cues. What we do know about the mechanisms that govern cell migration only serves to underscore the complexity of the system, particularly in cases where there is more than one input signal. By increasing our comprehension of how cells can differentially integrate multiple signals to finely tune cell behavior, we may be able to gain greater control over complex cellular systems and address the discrepancies between what we know of cell migration and the actual experimental observations across different cell populations. Ultimately, this knowledge will facilitate the design and improvement of bioengineered scaffolds and aid in the development of more personalized and disease-specific treatments.
